# Assessment of oral health-related quality of life and periodontal state in patients with end-stage renal disease: a comparison study using propensity score-matched analysis

**DOI:** 10.4317/medoral.26714

**Published:** 2024-10-13

**Authors:** Eun-Young Kwon, Ji-Young Joo

**Affiliations:** 1 Dental Clinic Center, Pusan National University Hospital, Busan, South Korea; 2 Biomedical Research Institute, Pusan National University Hospital, Busan, South Korea; 3 Department of Periodontology, Dental and Life Science Institute, School of Dentistry, Pusan National University, Yangsan, South Korea

## Abstract

**Background:**

The purpose of this study was to examine the periodontal state and oral health-related quality of life (OHRQoL) of patients with end-stage renal disease (ESRD) and the effect of ESRD on OHRQoL by comparison with age- and sex-matched systemic healthy controls with similar periodontal status levels.

**Material and Methods:**

Fifty patients with ESRD and 150 healthy individuals without ESRD were recruited. Medical characteristics were recorded for the test group and periodontal examination was performed in both groups. A structured Korean version of the Oral Health Impact Profile (OHIP-14K) questionnaire for subjective evaluation of OHRQoL was administered. To evaluate the effect of ESRD on OHRQoL, 50 healthy controls who had been matched for age, sex, and periodontal status were selected and compared with ESRD patients by using propensity score-matched analysis.

**Results:**

ESRD patients had significantly higher mean score of Community Periodontal Index and mean numbers of missing teeth compared with the controls (*P* < 0.05). Mean OHIP-14K total score and mean scores of all subdomains were significantly higher in the test group than in the controls (*P* < 0.05). Even after matching analysis, mean OHIP-14K total score and mean scores of subdomains were significantly higher in the test group than in the control group (*P* < 0.05), except in one subdomain (physical pain).

**Conclusions:**

Within the limitations of this investigation, the present study showed worse periodontal state and lower level of OHRQoL in ESRD patients compared with those in healthy subjects. Through comparison with matched healthy controls, ESRD was found to have a negative effect on OHRQoL.

** Key words:**Kidney diseases, oral health, periodontitis, quality of life.

## Introduction

Chronic kidney disease (CKD) is a public health problem that is accompanied by continual reduction of renal function, and often causes progression to end-stage renal disease (ESRD) (1). In the early-stage renal disease, many compensatory mechanisms help to maintain homeostasis of renal function (2). However, once the glomerular filtration rate (GFR) declines below the range of 10-20 mL/min/1.73 m2 body surface area with various complications in the CKD state, ESRD follows (3). Patients with ESRD and irreversible renal damage might have complex medical issues centered in the metabolic-endocrine, hematologic, immunologic, cardiovascular, or neuromuscular system (4). Diabetic nephropathy, glomerulonephritis, interstitial nephritis, hypertension, vascular disease, obstructive uropathy, autoimmune disease, and obesity are common causes of ESRD (5).

Studies have shown that up to 90% of patients with ESRD present with one or more of several clinical oral findings, such as xerostomia, gingival enlargement, mucosal lesions, impaired wound healing, alveolar bone destruction, and various oral infections (6). ESRD is associated with alterations in the salivary composition and flow rate leading to increased calculus deposition and consequent progression to gingival inflammation (7). Additionally, patients with ESRD are less likely to provide requisite attention on oral health maintenance because of the time-consuming renal treatment, including dialysis and the associated severe psychological burden (8). Negligence of oral hygiene with unfavorable periodontal condition in ESRD patients often causes periodontal infection, leading to the formation of periodontal pockets of the destroyed periodontium and eventual loss of natural teeth (9). Periodontal infection can cause pain and discomfort in chewing followed by degradation of oral health-related quality of life (OHRQoL), which is an element of overall health-related quality of life (10).

Although many researchers have evaluated periodontal status and OHRQoL in patients with ESRD (11-14), a comparative analysis with healthy controls was performed in few studies. Among the studies that compared the periodontal status and OHRQoL of ESRD patients with those of healthy controls (13,14), few compared the conditions with a matched control group, especially regarding OHRQoL. Furthermore, a previous study reported a clear association between periodontitis severity and OHRQoL in ESRD patients (12). The study showed a negative impact of periodontitis on OHRQoL in ESRD patients, but these results were not derived by comparing with a matched control (12). Based on these results, as periodontal status can affect OHRQoL in ESRD patients, the differences in OHRQoL between ESRD patients and healthy individuals with similar periodontal status levels and the influence of ESRD on OHRQoL should be investigated. Accordingly, the objectives of the present investigation were to examine the periodontal state and OHRQoL of ESRD patients compared with those of healthy controls, as well as the effect of ESRD on OHRQoL by comparison with age- and sex-matched systemic healthy controls with similar periodontal status levels.

## Material and Methods

- Study population

This cross-sectional study, conducted between March 2016 and December 2022, enrolled patients who visited the dental clinic of Pusan National University Hospital (PNUH), Korea. Patients who had been diagnosed with CKD at least 90 days prior and followed up on by the Department of Nephrology were referred to the Department of Periodontology for evaluation of their periodontal status. For each patient, the results of their laboratory tests were collected from the electronic medical records at the time of their periodontal examination. The mean values of the C-reactive protein (CRP) and estimated GFR (eGFR) levels were recorded. CRP, which is the major serum component of the acute-phase response (15), was assessed for identification of systemic inflammation. GFR, which is considered to be the gold standard for evaluation of renal function, was measured by serum creatinine concentration and calculated according to following equation (16):

eGFR (mL/min/1.73 m2) = 175 x (serum creatinine concentration)-1.154 x (age in years)-0.203 x (0.742 if female) x (1.210 if African-American).

Among the patients with CKD, 50 who exhibited CKD grade 5 (< 15 eGFR mL/min/1.73 m2) according to the criteria of the Kidney Disease: Improving Global Outcomes (KDIGO) clinical practice guidelines (17) and had been diagnosed as ESRD by nephrologists were selected for the test group. We excluded patients who had any systemic diseases that could acutely affect the kidney state, such as rapid progressive glomerulonephritis, active glomerular diseases, pregnancy, immunodeficiency syndrome, or any cancer.

**Figure 1:**
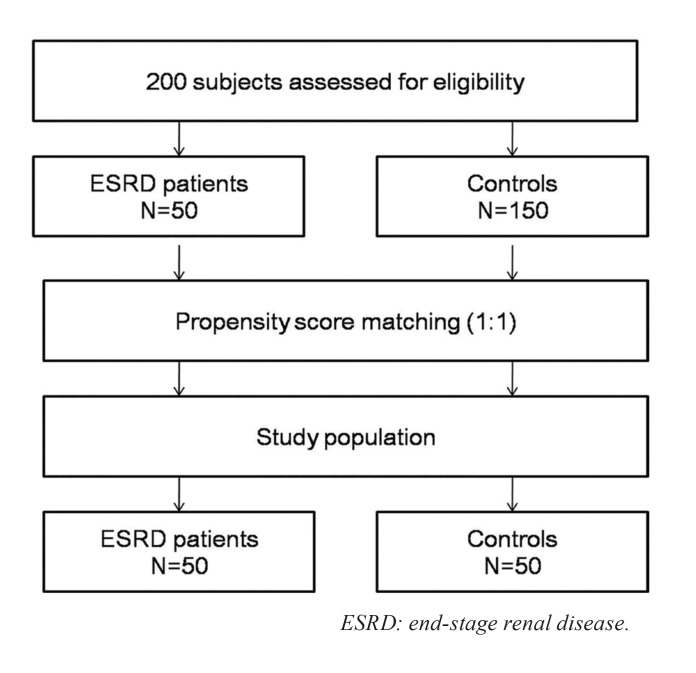
Flow chart of the subjects enrolled in the present study.

A total of 150 adults older than 20 years who had no disqualifying medical history were recruited for the purposes of comparison (the control group). To evaluate the effect of ESRD on OHRQoL, we selected age- and sex-matched healthy controls with similar periodontal status levels according to propensity score-matching analysis. Additionally, a subgroup of control individuals matched to the individuals in the test group was formed according to the propensity score values. This allowed for comparison of age- and sex-matched individuals with similar periodontal status levels but dissimilar OHRQoL scores. Therefore, a total of 50 healthy controls were finally included in this study (Fig. 1).

A sample size was calculated using a software program (G*Power software version 3.1, Heinrich Heine University Düsseldorf, Dusseldorf, Germany). Based on a significance level of 0.05, power of 80%, and 1:3 ratio of cases to controls, at least 127 healthy controls and 43 individuals with ESRD were required in this study.

All participants completed an informed consent form prior to the study. The study protocol was reviewed and approved by the Ethics Committee of PNUH (PNUH E-2016022) and conducted in accordance with the Helsinki Declaration of 1975 (revised in 2013).

- Study protocol

Demographic assessments: A single calibrated investigator gathered information on demographic variables, systemic health state, as well as the type and duration of renal therapy and recorded it.

Periodontal examination: For assessment of periodontal status, the Community Periodontal Index (CPI) was measured for both groups at six sites per tooth (mesio-buccal, mid-buccal, disto-buccal, disto-lingual, mid-lingual, mesio-lingual) using a periodontal probe. The CPI score was calculated according to the WHO protocol (18) and coded as follows:

Code 0—healthy periodontium

Code 1—bleeding on gentle probing

Code 2—calculus deposition

Code 3—probing depth of 4 to 5 mm

Code 4—probing depth of 6 mm or deeper.

The index teeth in each sextant to be evaluated were #17, 16, 11, 26, 27, 37, 36, 31, 46, and 47. If the index teeth were absent, all of the remaining teeth in that sextant were assessed, and the highest score was recorded. The highest CPI code among all six sextants was recorded as the patient’s final CPI score representative of his periodontal condition. According to the WHO definition of periodontitis, if a subject has a CPI score of 3 or 4, he is diagnosed with periodontal disease. The number of missing teeth was also measured for each individual.

All clinical data were collected by one calibrated periodontist at the PNUH Dental Clinic center. Before the study began, the intra-examiner reliability of the investigator was confirmed by measuring the CPI scores of 20 subjects repeatedly within a 1-week interval (intra-class correlation coefficient: 0.97).

Questionnaire: Subjective evaluation of OHRQoL was tested by the Korean version (translated and validated by K.H. Bae (19)) of the Oral Health Impact Profile (OHIP-14K) questionnaire (20). The OHIP-14K questionnaire’s 14 questions evaluated the following seven subdomains of oral health: functional limitation, physical pain, psychological discomfort, physical disability, psychological disability, social disability, and handicap. The answers were scored on a 5-point Likert scale as follows:

0, never; 1, hardly ever; 2, occasionally; 3, fairly often; 4, very often. The OHIP-14K total score, calculated as the sum of the 14 questions, ranged from 0 to 56, higher scores indicating poorer OHRQoL. The internal consistency of each questionnaire was evaluated according to Cronbach’s alpha, which was 0.95, as indicative of the high internal consistency of OHIP-14.

- Statistical analysis

The data were evaluated using the SPSS statistical software program (IBM, Armonk, NY, USA). Descriptive statistics, such as means and standard deviations were used to characterize the subjects. To evaluate the effect of ESRD on OHRQoL, the balance of the differences in the baseline characteristics between the test and control groups was required. To balance the two groups, the propensity scores were calculated using a logistic regression model and the following covariates: age, sex, CPI score, and number of missing teeth. Based on the propensity score values, a subgroup of control individuals was formed and 1:1 matched to individuals in the test group. The Kolmogorov-Smirnov test was used to assess the normality of the distributions of the variables. For comparison of the differences between the test and control groups, data were analyzed by the independent t-test for normally distributed continuous variables and by the Mann-Whitney U test for non-normally distributed continuous variables. The chi-square test was applied for comparisons between the categorical variables. Statistical significance was set at *P* < 0.05.

## Results

All patients in the test group underwent dialysis for ESRD. The number of patients who underwent hemodialysis (*n*=34, 68%) was significantly higher than that of patients on peritoneal dialysis (*n*=16, 32%). The mean dialysis duration was 4.66 ± 2.90 years (range:1 - 12 years). The mean eGFR and CRP values in the test group were 6.77 ± 0.45 mL/min/1.73 m2 (range: 3.10-14.70 mL/min/1.73 m2) and 0.96 ± 3.17 mg/dL (range: 0.00-20.57 mL/min/1.73 m2), respectively.

The participants’ demographic characteristics and periodontal status are provided in Table 1. The inter-group mean age and female distribution were significantly different before propensity score matching (*P* < 0.05). Additionally, the patients with ESRD had a significantly higher mean CPI score and a greater mean number of missing teeth compared with the controls before matching (*P* < 0.05). To evaluate the effect of ESRD on OHRQoL, propensity score matching analysis was performed. After matching, it was confirmed that there were no significant differences in age, sex, and periodontal status between the test group and the control group.

**Figure 2:**
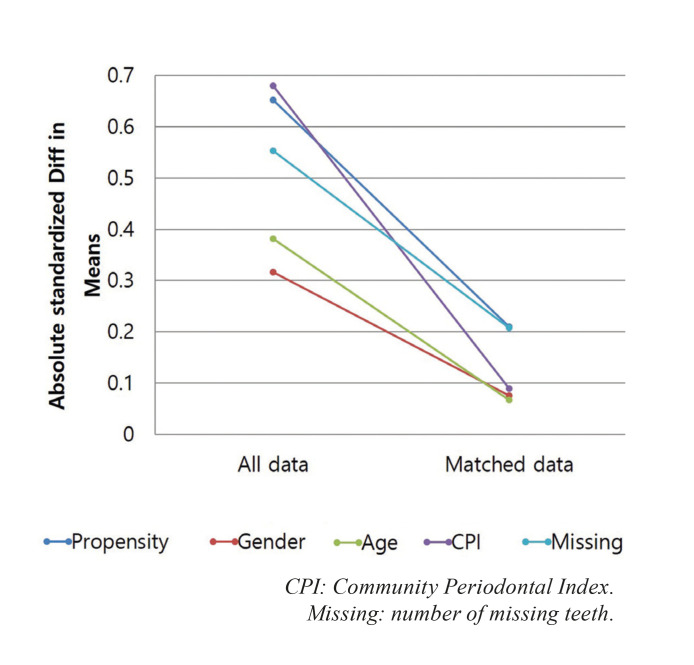
Absolute standardized mean differences in data before and after propensity score matching. After matching, absolute standardized mean differences of all covariates were reduced to 0.25.

As shown in Table 1, the mean OHIP-14K total score and mean scores of all subdomains were significantly higher in the test group than in the control group before matching (*P* < 0.05). Even after matching analysis, the mean OHIP-14K total score and mean scores of subdomains were significantly higher in the test group than in the control group (*P* < 0.05), except for one subdomain (physical pain). Additionally, mean differences and 95% confidence interval between the groups before and after propensity score matching (1:1 matching) are provided in Supplement 1. The absolute standardized mean differences of all covariates after propensity score matching were reduced to < 25% (Fig. 2).

## Discussion

Many studies have proposed that periodontitis is a potential risk factor for various systemic diseases, such as atherosclerosis, diabetes mellitus, rheumatoid arthritis, and renal insufficiency (21). Although periodontitis is a local infection of the oral tissue, it is a novel potential risk factor for kidney dysfunction and a possible contributing factor to the inflammatory burden in CKD (22). Periodontal pathogens can trigger a tissue-destructive immune-inflammatory response associated with systemic dissemination of periodontal bacteria and their inflammatory products (23). Through an inflammatory pathway, periodontal pathogenic bacteria can contribute to the overall systemic inflammatory burden and affect even distant organs, such as the kidneys (23).

Periodontitis is related to increased CRP levels resulting from the acute-phase response to inflammation (24). CRP is a sensitive serological marker of inflammation, and has been posited as a mediator of the relationship between periodontitis and systemic illness (25). Increased CRP levels might facilitate more intense local CRP deposition and amplify subseqent inflammatory buderns, resulting in various complications (22). Interestingly, previous studies have found that only initial periodontal therapy, such as local scaling and root planning may reduce the serum levels of some pro-inflammatory biomarkers (26). The accumulated data indicate that periodontitis in ESRD patients is a potential risk factor for increased serum CRP levels, and, moreover, that effective periodontal treatment can decrease serum CRP levels (27). In the present study, the mean CRP level of the ESRD patients was higher than normal. Although it cannot be concluded that periodontitis in the ESRD patients was a major contributing factor to their increased level of serum CRP, their inflammatory periodontal status may lead to increased levels of CRP.

CKD also can affect the oral tissues and induce many problems, such as increased levels of plaque, calculus, and possible periodontal inflammation. Such problems are known to be correlated with elevated status of systemic inflammation and the cumulative effect of negligence of oral hygiene over the course of years in CKD patients (27). Additionally, CKD often leads to progressive and irreversible reduction of GFR followed by ESRD. Many ESRD patients are in a state of uremia that is accompanied by immune dysfunction because of impaired function of lymphocytes, monocytes, and macrophages (28). This can result in diminished host defense against the subgingival microbial challenge and increased prevalence or severity of gingivitis and periodontitis (7). In the present study, the ESRD patients showed a significantly higher mean CPI score and a greater mean number of missing teeth when compared with those of the healthy individuals. Although it is difficult to determine whether such poor periodontal status was caused exclusively by ESRD, it can be assumed that ESRD, with negligence of oral hygiene care owing to long-term renal therapy, is a potential contributing factor to inflammatory periodontal status. Based on these findings, the periodontal status of ESRD patient needs to be carefully monitored for improvement of their periodontal status and reduction of the systemic inflammatory burden. Chen and Li (29) reported that periodontal treatment could reduce the risk of mortality in ESRD patients with periodontitis. Therefore, to enhance the periodontal and systemic status in ESRD patients, reinforcement of oral hygiene care, supportive periodontal treatment, and instructions on the necessity of regular dental visits in addition to renal therapy should be emphasized.

OHRQoL is a multi-dimensional concept and an essential element of overall health-related quality of life (14). When considering the poor periodontal status of ESRD patients compared with that of healthy subjects, degradation of OHRQoL is an expected consequence. However, some studies reported that ESRD had no important effect on OHRQoL (11,14). Guzeldemir *et al*. (11) found that the impact of ESRD on OHRQoL was moderate, implying that oral health was not a major concern in ESRD patients. The researchers found that ESRD patients thought their poor oral condition was simply a complication of CKD and did not pay much attention to their oral health status. Schmalz *et al*. (14) also showed neither clinically relevant nor statistically significant differences in OHIP-14 scores between ESRD patients and healthy controls. They concluded that this was because ESRD patients focused on their overall systemic health and were relatively insensitive to their oral health status.

In contrast, some researchers proposed that ESRD patients reported significantly poorer OHRQoL in comparison that of healthy controls (13,30). This is consistent with the findings of our study, which reported poorer OHRQoL in ESRD patients. In the present study, the mean total OHIP-14K score and mean subdomain scores were significantly higher for the ESRD patients than for the controls before matching. This may be associated with a higher CPI score and greater number of missing teeth, which can induce gingival bleeding, tooth mobility, and discomfort during mastication in ESRD patients. It is thought that such oral discomfort may have had a negative impact on OHRQoL.

In this study, we not only calculated the total OHIP-14K score and subdomain score in each group but also compared age- and sex-matched healthy controls who had similar periodontal status levels to evaluate the effects of ESRD on OHRQoL. The mean total OHIP-14K score and mean subdomain scores were significantly higher for the ESRD patients than for the healthy controls even after propensity score matching, except for one subdomain (physical pain). This result may support that ESRD has a negative impact on OHRQoL. Therefore, clinicians should recognize that OHRQoL could be reduced in ESRD patients and attempt to improve both physical and psychological problems in these patients.

The lack of statistically significant difference in physical pain among the subdomains of OHIP-14 after matching analysis may have been because ESRD patients were likely to be accustomed to chronic pain. In this study, the mean duration of dialysis within the ESRD group was over 4 years and the patients might have become less sensitive to pain because of the long treatment period.

The main limitation of the current study is its cross-sectional approach, which incurred difficulty in determining the exact cause-and-effect relationship between periodontitis and ESRD. Further longitudinal studies with sufficient follow-up periods are recommended to acquire additional and more detailed information on the causative association between periodontal status and ESRD. Additionally, this study did not consider other potential contributing factors, such as oral-health behavior, underlying systemic diseases, and socioeconomic status. Future larger-sample-size and interventional studies regarding these factors are necessary to determine whether they could influence the results of the present study.

Within the limitations of this investigation, the ESRD patients showed poorer periodontal state and lower level of OHRQoL compared with those of healthy individuals. Through comparison with matched healthy controls, ESRD was found to have a negative effect on OHRQoL. This result suggests that ESRD is a potential risk factor for periodontal health and OHRQoL. Therefore, adequate periodontal therapy to reduce localized and systemic inflammation and any attempt to improve OHRQoL should be considered to be an integral part of ESRD patients’ comprehensive treatment.

## Figures and Tables

**Table 1:** Demographic characteristics and descriptive statistical values before and after propensity score matching (1:1 matching).

Variables	Before matching			After matching		
	Test group (n=50)	Control group (n=150)	*P-value*	Test group (n=50)	Control group (n=50)	*P-value*
Age (years)	52.64± 12.90	47.71± 12.58	0.018*^a^	52.64 ± 12.90	51.86 ± 10.34	0.975^b^
Sex Female/Male (female %)	25/25 (50.0%)	99/51 (66.0%)	0.044*^c^	25/25 (50.0%)	27/23 (54.0%)	0.689^c^
CPI score	3.54 ± 0.81	2.99 ± 1.26	0.008*^b^	3.54 ± 0.81	3.48 ± 0.97	0.982^b^
Number of missing teeth	2.24 ± 2.87	0.65 ± 1.34	<0.001*^b^	2.24 ± 2.87	1.64 ± 1.90	0.470^b^
OHIP-14K Total	25.66 ± 8.57	12.43 ± 9.56	<0.001*^b^	25.66 ± 8.57	14.46 ± 9.67	<0.001*^b^
Functional limitation	3.10 ± 1.68	0.99 ± 1.50	<0.001*^b^	3.10 ± 1.68	1.18 ± 1.42	<0.001*^b^
Physical pain	3.58 ± 1.75	2.51 ± 2.01	0.001*^b^	3.58 ± 1.75	2.90 ± 2.23	0.106^b^
Psychological discomfort	4.00 ± 1.69	2.25 ± 1.75	<0.001*^b^	4.00 ± 1.69	2.58 ± 1.73	<0.001*^b^
Physical disability	3.90 ± 2.09	2.03 ± 2.09	<0.001*^b^	3.90 ± 2.09	2.60 ± 2.18	0.002*^b^
Psychological disability	3.70 ± 1.83	1.60 ± 1.73	<0.001*^b^	3.70 ± 1.83	1.68 ± 1.46	<0.001*^b^
Social disability	3.58 ± 2.01	1.61 ± 1.77	<0.001*^b^	3.58 ± 2.01	1.76 ± 1.69	<0.001*^b^
Handicap	3.80 ± 1.86	1.45 ± 1.62	<0.001*^b^	3.80 ± 1.86	1.76 ± 1.74	<0.001*^b^

Values are the mean ± standard deviation; ^a^*P-value* determined by independent t-test; ^b^ *P-value* determined by Mann-Whitney U test; ^c^ *P-value* determined by chi-square test; *Statistically significant, P < 0.05; Abbreviations: CPI, Community Periodontal Index. OHIP-14K, Korean version of Oral Health Impact Profile-14.
